# Patient-Reported Symptoms Versus Clinician-Measured Signs to Distinguish Sjogren's in Patients With Dry Eye

**DOI:** 10.1167/tvst.15.1.27

**Published:** 2026-01-22

**Authors:** Michael X. Lin, Julia Zeng, David Cui, Lee W. Guo, Gavin Li, Kyle Munar, Michela Montecchi-Palmer, Ian J. Saldanha, Esen K. Akpek

**Affiliations:** 1 The Ocular Surface Disease Clinic, The Wilmer Eye Institute, The Johns Hopkins University School of Medicine, Baltimore, MD, USA; 2 University of Maryland School of Medicine, Baltimore, MD, USA; 3 Stritch School of Medicine, Loyola University, Chicago, IL, USA; 4 Sinai Hospital of Baltimore, Baltimore, MD, USA; 5 Department of Ophthalmology, Massachusetts Eye and Ear Infirmary, Harvard Medical School, Boston, MA, USA; 6 Alcon Inc., Fort Worth, TX, USA; 7 Johns Hopkins University Bloomberg School of Public Health, Baltimore, MD, USA

**Keywords:** Sjogren's disease, dry eye disease, differential diagnosis

## Abstract

**Purpose:**

The purpose of this study was to evaluate clinical factors that could differentiate patients with Sjogren's disease-related dry eye (SDDE) from non-SDDE.

**Methods:**

Patients with SDDE (*n* = 20), non-SDDE (*n* = 47), and healthy control participants (*n* = 29) were enrolled. Patients with dry eye were required to have physician-diagnosed dry eye disease for at least 6 months prior to the study. Sjogren's diagnosis was made according to 2016 Sjogren's disease criteria. Control participants had no known dry eye diagnosis or ocular and/or autoimmune disease. Several validated questionnaires regarding overall health and perception of health, mental status, review of systems, and ocular symptom were administered concurrently. Ocular surface and tear film parameters were evaluated using strict methodology.

**Results:**

Each questionnaire, each section within a questionnaire, and each question within a section were evaluated. Of systemic symptom questionnaires, only Profile of Fatigue and Discomfort Questionnaire (PROFAD) differentiated between SDDE and non-SDDE (10.5 vs. 5.8, *P* = 0.003). Among four Sjogren's Foundation recommended review of systems questions, only dry mouth differentiated patients with SDDE from non-SDDE (90% vs. 53%, *P* = 0.004). No mental health or ocular symptom questionnaires were different between SDDE versus non-SDDE. Among clinician-measured ocular surface parameters, only corneal fluorescein (2.5 vs. 1.0, *P* = 0.006) and conjunctival lissamine green (2.0 vs. 1.0, *P* = 0.001) staining scores were significantly worse in patients with SDDE compared to non-SDDE.

**Conclusions:**

Ocular staining and dry mouth complaints were strongly associated with SDDE.

**Translational Relevance:**

Ocular surface vital dye staining and inquiring about dry mouth complaints can be helpful to determine whether Sjogren's laboratory work-up should be considered in patients presenting with dry eye disease.

## Introduction

Dry eye disease is a common inflammatory ophthalmic condition, affecting an estimated 5% to 15% of the United States adult population.^1^^–^^3^ It is associated with poor quality of life and impaired daily function due to ocular discomfort symptoms and blurred or fluctuating vision.^4^^–^^6^ Sjogren's disease is a chronic autoimmune condition associated with a progressive form of dry eye leading to ocular complications, including vision-threatening corneal disease.^7^^–^^12^ Approximately 30% to 40% of patients with Sjogren's disease also develop systemic extraglandular manifestations such as arthritis, myositis, vasculitis, interstitial lung or kidney disease, neuropathy, and particularly lymphoma.^12^^–^^15^ Approximately 98% of all patients with Sjogren's disease have dry eye disease and approximately 10% of all patients with clinically significant dry eye disease have an underlying Sjogren's disease.^6^^,^^11^^,^^16^^,^^17^ Yet, currently, only approximately 5% of patients with dry eye are referred for a laboratory workup to uncover an underlying Sjogren's disease.^18^ Further, these referrals are largely based on severity of subjective patient-reported ocular symptoms.^18^ This continuing under-referral results in underdiagnosis and delay in diagnosis of Sjogren's disease by a decade with the potential for systemic and ocular morbidity.^6^^,^^11^^,^^19^^–^^21^ Accurate timely diagnosis of Sjogren's disease and identifying the most relevant characteristics of Sjogren's disease-related dry eye (SDDE) is a current area of clinical and research focus.

The purpose of our study was to assess the usefulness of various symptom questionnaires and clinical parameters in patients with dry eye disease, in order to identify the most useful and practical evaluations to differentiate patients with SDDE from patients with non-SSDE in real-world practice.

## Methods

We conducted an observational cross-sectional study at the Ocular Surface Disease Clinic of The Wilmer Eye Institute, The Johns Hopkins University School of Medicine. The University Institutional Review Board provided ethical approval for the study (IRB00247104). We obtained written informed consent from all study participants before their participation. The study adhered to the tenets of the Declaration of Helsinki.

### Participants

Study participants were recruited from a clinic-based sample of adult (≥18 years) male or female patients with dry eye disease diagnosis made by an eye care specialist at least 6 months prior to presentation. Control participants were age-matched accompanying family members or friends with no history of dry eye, ocular surface diseases, or autoimmune conditions. Patients with other systemic conditions that could interfere with ocular surface disease evaluations, such as diabetes, neurological conditions, or patients who were on medications that could cause ocular surface dryness, were also excluded. All eligible participants were required to have a visual acuity of 20/40 in each eye. Diagnosis of SDDE was established according to the 2016 Sjogren's disease criteria,^22^ which require at least one laboratory finding (positive serology – anti-SSA antibody or positive minor salivary gland biopsy – focal lymphocytic sialadenitis with a focus score ≥1 foci/mm^2^) and at least one clinical finding (dry eye [combined corneal and conjunctival staining score ≥5 in at least one eye, or unanesthetized Schirmer tear wetting ≤5 mm/5 minutes in at least one eye], or dry mouth [unstimulated salivary flow rate ≤0.1 mL/min]).^22^ Participants with non-SDDE had a negative minor salivary gland biopsy as well as negative serology. Control participants were required to have no history of dry eye or any underlying autoimmune disease, no significant dry eye symptoms (Ocular Surface Disease Index [OSDI] <13) and normal tear production and no ocular surface vital dye staining. The details of methodology for testing for tear film and ocular surface parameters are provided below.

Exclusion criteria included literacy limitations, contact lens use within 10 days before the visit, intraocular surgery within 3 months before the visit, minor ocular surgery or eyelid procedures within 30 days before the visit, corneal surgery or cosmetic lid surgery within 12 months before the visit, any history of glaucoma requiring topical treatment or previous glaucoma surgery, visual acuity worse than 20/40, current smoker status or pregnancy, or artificial tear use within 2 hours before the visit. We also excluded individuals with a history of hepatitis C, HIV, sarcoidosis, amyloidosis, graft versus host disease, cicatrizing conjunctivitis, pemphigoid, drug-induced pseudo-pemphigoid, chemical burns, atopic keratoconjunctivitis, mucous membrane pemphigoid, neurotrophic keratoconjunctivitis, or ocular scarring due to viral etiology. Additionally, non-SDDE patients and control participants were excluded if they had history of any autoimmune, inflammatory, or rheumatologic disease or any disease (e.g. diabetes, neurological disease, and cosmetic chemodenervation injections) or medication (e.g. antihistamines and diuretics) that could potentially interfere with ocular surface and tear film evaluations.

### Visit Schedule and Outcome Assessments

Information regarding each participant's demographic information, prior medical and ocular diagnoses, and concomitant medications was collected. Participants then completed several previously validated questionnaires that are commonly used in the field of Sjogren's disease to assess mood, overall health, and perception of health and fatigue and pain (Supplementary Appendix A).

Mental health of participants was assessed by administering the Mood Questionnaire (a 7-item questionnaire that assesses mood adapted from the Columbia Suicide Severity Rating Scale and Beck's Depression Inventory, range = 0–21),^23^^,^^24^ Mini Mental State Examination (MMSE; an 11-item questionnaire that screens for cognitive impairment, range = 0–30),^25^ and Center for Epidemiologic Studies-Depression Questionnaire (CES-D; a 20-item questionnaire that screens for major depressive disorder, range = 0–60).^26^

Overall health and perception of the participants’ health was assessed by administering the 36-Item Short Form Survey (SF-36; a 36-item questionnaire that assesses general health in 8 domains: physical functioning, physical health, bodily pain, general health, vitality, social functioning, emotional well-being, and mental health, range = 0–100),^27^ Profile of Fatigue and Discomfort Questionnaire (PROFAD; a 19-item questionnaire that measures eight domains of Sjogren's disease-related symptoms: somatic fatigue, mental fatigue, arthralgia, vascular dysfunction, and oral, ocular, cutaneous, and vaginal dryness, range = 0–133),^28^ European League Against Rheumatism (EULAR) Sjogren’s Syndrome Patient Reported Index (ESSPRI; a 3-item questionnaire that assesses dryness, fatigue, and joint/muscle pain, range = 0–30),^29^ and the Sjogren's Foundation recommended short review of systems (pain, fatigue, dry mouth, and family history of autoimmune disease, range = present or absent).^30^

Patient-reported ocular symptoms were assessed using the OSDI (a 12-item questionnaire that measures severity of dry eye symptoms and vision-related functioning, and environmental factors, range = 0–100),^31^ Impact of Dry Eye on Everyday Life (IDEEL; a 57-item questionnaire that assesses dry eye symptoms and their impact on daily living, range = 0–100),^32^ National Eye Institute-Visual Functioning Questionnaire (NEI-VFQ-25; a shortened version of the 51-item NEI-VFQ that measures several aspects of quality of life related to vision, range = 0–100),^33^ Visual Analogue Scales (VAS) for eye fatigue (VFAS) and eye dryness (VDAS; quick, repeatable indicators of the severity of dry eye, range = 0–100),^34^ and Visual Tasking Questionnaire (VTQ; a 14-item questionnaire that measures the extent to which vision-related activities are limited by the eye condition in the past 7 days, range = 13–52; Modus Outcomes, Cambridge, MA).

Clinical findings were assessed in the following order: distance-visual acuity using habitual correction (Snellen chart), contrast sensitivity (Mars letter contrast sensitivity test; The Mars Perceptrix Corporation, Chappaqua, NY), tear osmolarity (TearLab, San Diego, CA), Schirmer's test without anesthesia at 5 minutes (TearFlo; Keeler, Malvern, PA), corneal punctate erosion score using fluorescein (BioGlo, Farmington Hills, MI), and conjunctival staining score using lissamine (GreenGlo, Farmington Hills, MI). Corneal and conjunctival staining were graded based on both the Sjögren’s International Collaborative Clinical Alliance (SICCA) Ocular Staining Score system^35^ and the National Eye Institute^36^ (NEI) scales. SICCA corneal staining (range = 0–6) is based on the density of the punctate epithelial erosions (range = 0–3) plus one extra point for central, confluent, or filament staining. SICCA conjunctival staining (range = 0–6) is based on density (range = 0–3) of nasal and temporal conjunctival lissamine dye take up within the interpalpebral fissure. NEI corneal staining (range = 0–15) was scored by adding the punctate epithelial erosions grade (range 0–3) from five regions of the cornea (central, superior, inferior, temporal, and nasal). NEI conjunctival staining (range = 0–18) was scored by adding the density (range = 0–3) of lissamine green dye take up from six regions of the conjunctiva.

### Statistical Analysis

Demographics, questionnaire responses, and clinician-evaluated signs were compared among all three groups (SDDE, non-SDDE, and control) as well as between the two groups with dry eye (SDDE and non-SDDE). Pearson's χ^2^ tests were used to compare categorical variables, such as proportions of participants with specific demographic characteristics or participants reporting systemic Sjogren's symptoms, and Kruskal-Wallis test was used to compare medians among groups. The *P* < 0.05 was considered as statistically significant, and all analyses were conducted using Stata version 18.5 (StataCorp LLC, College Station, TX).

## Results

Table 1 displays the demographic characteristics of the study participants. Ninety-six participants (SDDE = 20, non-SDDE = 47, and control = 29) were enrolled between January 7, 2021, and October 3, 2022. Fewer control participants than participants with SDDE and participants with non-SDDE were older than 60 years (24% vs. 50% vs. 55%, *P* = 0.047) and women (62% vs. 95% vs. 72%, *P* = 0.03). The comparison of female percentages between the two groups with dry eye was also significantly different (*P* = 0.04) with more of the patients with SDDE being women. Most participants were White (71% of all participants) and non-Hispanic (93% of all participants). There were no statistically significant racial or ethnic differences among the groups.

**Table 1.** Demographic Characteristics of Participants in a Prospective, Observational Clinical Study to Distinguish Presence of Sjogren's in a Clinic-Based Sample of Patients With Dry Eye

					*P* Value	
Characteristic	SDDE (*n* = 20) *n* (%)	Non-SDDE (*n* = 47) *n* (%)	Controls (*n* = 29) *n* (%)	All (*n* = 96) *n* (%)	SDDE vs. Non-SDDE	All
Age, y *n* (%)						
18 to 40	1 (5)	8 (17)	6 (21)	15 (16)	0.24	**0.047**
41 to 60	9 (45)	13 (28)	16 (55)	38 (40)		
>60	10 (50)	26 (55)	7 (24)	43 (45)		
Sex, *n* (%)						
Female	19 (95)	34 (72)	18 (62)	71 (74)	**0.04**	**0.03**
Male	1 (5)	13 (28)	11 (38)	25 (26)		
Race,^a^ *n* (%)						
American Indian or Alaskan Native	0 (0)	0 (0)	0 (0)	0 (0)	0.28	0.40
Asian or Pacific Islander	3 (15)	2 (4)	2 (7)	7 (7)		
Black or African American	4 (20)	6 (13)	8 (28)	18 (19)		
White	13 (65)	37 (79)	18 (62)	68 (71)		
Other	0 (0)	2 (4)	1 (3)	3 (3)		
Ethnicity, *n* (%)						
Hispanic	1 (5)	3 (6)	0 (0)	4 (4)	0.80	0.70
Not Hispanic	18 (90)	43 (91)	28 (97)	89 (93)		
Not known	1 (5)	1 (2)	1 (3)	3 (3)		

SDDE, Sjogren's disease related dry eye; Non-SDDE, non-Sjogren's disease related dry eye.

Statistically significant *P* values for Chi-square test of independence are in bold.

a Respondents could select more than one response.

Table 2 displays the patient-reported symptom systemic questionnaires. Compared with participants with SDDE and non-SDDE, control participants had significantly better (*P* = 0.0001 for each) perceived health status as indicated on the SF-36 (87.4 vs. 64.3 vs. 74.5), fewer Sjogren's disease-related symptoms indicated by the PROFAD questionnaire (2.5 vs. 10.5 vs. 5.8), and fewer Sjogren's disease-related symptoms indicated by the ESSPRI questionnaire (0.7 vs. 5.4 vs. 4.2). There were no differences among the three groups on the Mood Disorder Questionnaire (MDQ: 0 vs. 0 vs. 0), CES-D (8.5 vs. 9.0 vs. 6.0), and MMSE (25.5 vs. 26.0 vs. 25.0) questionnaires. When comparing the systemic symptoms among the two groups, although participants with SDDE tended to report more symptoms on the ESSPRI (5.4 vs. 4.2, *P* = 0.07) and the SF-36 (64.3 vs. 74.5, *P* = 0.13), the differences were not statistically significant. There was a significant difference between participants with SSDE versus non-SSDE using only the PROFAD questionnaire (10.5 vs. 5.8, *P* = 0.003). Regarding specific items on the PROFAD questionnaire (data not shown in Table 2), participants with SDDE reported worse symptoms with respect to almost all items (exhaustion [*P* = 0.03], lacking strength in muscles [*P* = 0.03], forgetting things [*P* = 0.02], limb discomfort [*P* = 0.01], uncomfortably cold hands [*P* = 0.04], vaginal dryness [*P* = 0.02]), bad breath [*P* = 0.04], and other mouth problems such as ulcers [*P* = 0.03]), with dry mouth/throat/nose being the most significant symptom (*P* = 0.0005).

**Table 2.** Usefulness of Patient-Reported Systemic Symptom Questionnaires to Distinguish Presence of Sjogren's in a Clinic-Based Sample of Patients With Dry Eye

						*P* Value	
Questionnaire	Score Interpretation Higher is:	SDDE (*n* = 20) Median [IQR]	Non-SDDE (*n* = 47) Median [IQR]	Controls (*n* = 29) Median [IQR]	All (*n* = 96) Median [IQR]	SDDE vs. Non-SDDE	All
MDQ	Worse	0 [0, 0]	0 [0, 1.0]	0 [0, 0]	0 [0, 0]	0.40	0.49
Range = 0–21							
CES-D	Worse	8.5 [3.5, 19.5]	9.0 [3.0, 16.0]	6.0 [3.0, 10.5]	8.0 [3.0, 13]	0.72	0.51
Range = 0–60							
SF-36	Better	64.3 [51.3, 77.7]	74.5 [57.1, 85.3]	87.4 [79.8, 92.4]	78.7 [63.2, 88.0]	0.13	**0.0001**
Range = 0–100							
PROFAD	Worse	10.5 [8.4, 16.3]	5.8 [3.8, 10.3]	2.5 [1.5, 5.0]	5.6 [2.5, 9.9]	**0.003**	**0.0001**
Range = 0–133							
ESSPRI	Worse	5.4 [3.0, 6.8]	4.2 [2.3, 5.3]	0.7 [0, 1.3]	3.0 [1.0, 5.0]	0.07	**0.0001**
Range = 0–30							
MMSE	Better	25.5 [25.0, 26.0]	26.0 [25.0, 26.0]	25.0 [24.0, 26.0]	26.0 [25.0, 26.0]	0.56	0.48
Range = 0–30							

CES-D, Center for Epidemiologic Studies Depression Scale; ESSPRI, EULAR Sjogren’s Syndrome Patient Reported Index; IQR, interquartile range; MDQ, Mood Disorder Questionnaire; MMSE, Mini Mental Status Examination; non-SDDE, non-Sjogren's disease dry eye; PROFAD, Profile of Fatigue and Discomfort Questionnaire; SDDE, Sjogren's disease related dry eye; SF-36, The 36-Item Short Form Health Survey.

Statistically significant *P* values for Kruskal-Wallis test for medians are in bold.

Table 3 shows data for the Sjogren's Foundation recommended short review of systems. Compared with participants with SDDE and non-SDDE, a significantly lower percentage of control participants had joint paints (21% vs. 75% vs. 55%, *P* < 0.001), fatigue (10% vs. 60% vs. 38%, *P* = 0.001), and dry mouth (3% vs. 90% vs. 53%, *P* < 0.001). A larger percentage of participants with SDDE than non-SDDE and control participants had a family history of autoimmune disease, although this was not statistically significant (40% vs. 28% vs. 21%, *P* = 0.37). When comparing the groups of participants with dry eye, a significantly greater percentage of participants with SDDE had dry mouth (*P* = 0.004).

**Table 3.** Usefulness of Sjogren's Foundation Recommended Short Review of Systems Questions to Distinguish Presence of Sjogren's Disease in a Clinic-Based Sample of Patients With Dry Eye

					*P* Value	
Characteristic	SDDE (*n* = 20) *n* (%)	Non-SDDE (*n* = 47) *n* (%)	Controls (*n* = 29) *n* (%)	All (*n* = 96) *n* (%)	SDDE vs. Non-SDDE	All
Joint pain					0.13	**<0.001**
Yes	15 (75)	26 (55)	6 (21)	49 (49)		
No	5 (25)	21 (45)	23 (79)	47 (51)		
Fatigue					0.10	**0.001**
Yes	12 (60)	18 (38)	3 (10)	33 (34)		
No	8 (40)	29 (62)	26 (90)	63 (66)		
Dry mouth					**0.004**	**<0.001**
Yes	18 (90)	25 (53)	1 (3)	44 (46)		
No	2 (10)	22 (47)	28 (97)	52 (54)		
Family history of autoimmune disease					0.32	0.37
Yes	8 (40)	13 (28)	6 (21)	27 (28)		
No	12 (60)	34 (72)	22 (79)	68 (72)		

Statistically significant *P* values for Chi-square test of independence are in bold.

Table 4 displays the median scores of ocular symptom questionnaires. Compared with participants with SDDE and non-SDDE, control participants reported significantly fewer dry eye symptoms as indicated by the OSDI (6.3 vs. 29.2 vs. 31.5, *P* = 0.0001) and the IDEEL (77.7 vs. 60.3 vs. 65.3, *P* = 0.0001), less eye dryness (3.0 vs. 60.0 vs. 49.0, *P* = 0.0001), and eye fatigue (4.0 vs. 49.0 vs. 49.5, *P* = 0.0001) by the VAS, better vision-related quality of life, as indicated by the NEI-VFQ-25 (97.3 vs. 84.1 vs. 88.3, *P* = 0.0001), and fewer vision-related difficulties when performing tasks as indicated by the VTQ (14.0 vs. 21.5 vs. 18.0, *P* = 0.0001). The two groups with dry eye had similar scores for all ocular symptom questionaries. When analyzing individual questions and each sub-section on ocular symptom questionnaires, no individual ocular symptom or subsection definitively differentiated between participants with SDDE and non-SDDE.

**Table 4.** Usefulness of Patient-Reported Ocular Symptom Questionnaires to Distinguish Presence of Sjogren's Disease in a Clinic-Based Sample of Patients With Dry Eye

						*P* Value	
Questionnaire	Score Interpretation Higher Is:	SDDE (*n* = 20) Median [IQR]	Non-SDDE (*n* = 47) Median [IQR]	Controls (*n* = 29) Median [IQR]	All (*n* = 96) Median [IQR]	SDDE vs. Non-SDDE	All
OSDI	Worse	29.2 [14.8, 52.1]	31.5 [14.2, 42.7]	6.3 [2.1, 10.9]	18.8 [7.6, 38.5]	0.65	**0.0001**
Range = 0, 100							
IDEEL	Worse	60.3 [53.0, 72.2]	65.3 [55.0, 73.6]	77.7 [75.5, 82.8]	71.1 [58.3, 77.5]	0.51	**0.0001**
Range = 0, 100							
NEI-VFQ-25	Better	84.1 [77.1, 90.3]	88.3 [78.8, 91.4]	97.3 [94.8, 98.4]	90.5 [82.8, 96.2]	0.22	**0.0001**
Range = 0, 100							
*VFAS*	Worse	60.0[25.0, 74.5]	49.0 [14.0, 67.0]	3.0 [1.0, 10.0]	29.5 [6.0, 65.0]	0.23	**0.0001**
Range = 0, 100							
VDAS	Worse	49.0 [31.5, 74.0]	49.5 [23.0, 67.5]	4.0 [0.0, 7.0]	35.0 [6.0, 62.0]	0.66	**0.0001**
Range = 0, 100							
VTQ	Worse	21.5 [14, 29.5]	18.0 [14.25, 25.0]	14.0 [13.0, 15.0]	15.0 [13.0, 22.0]	0.28	**0.0001**
Range = 13, 52							

OSDI, Ocular Surface Disease Index; IDEEL, Impact of Dry Eye on Everyday Life Questionnaire; NEI-VFQ-25, 25-item National Eye Institute Visual Functioning Questionnaire; VFAS, Visual Fatigue Analogue Scale; VDAS, Visual Dryness Analogue Scale; VTQ, Visual Tasking Questionnaire.

Statistically significant *P* values for Kruskal-Wallis test for medians are in bold.

Table 5 displays the results of physician-measured ocular surface and tear film parameters. Compared with participants with SDDE and non-SDDE, control participants had higher tear production (17 vs. 8.5 vs. 11, *P* = 0.01), less ocular surface staining using both the SICCA (1 vs. 8 vs. 3, *P* = 0.0001) and the NEI (3 vs. 19 vs. 7, *P* = 0.0001) scoring systems, and higher tear break up time (TBUT; 7 vs. 4 vs. 4, *P* = 0.0001). When comparing the patients with dry eye, those with SDDE had significantly worse ocular surface staining scores using both the SICCA (*P* = 0.0001) and the NEI (*P* = 0.0001) scales. This difference remained significant when considering corneal as well as conjunctival (total and temporal and nasal separately) staining using either scoring systems. No significant differences in visual acuity, contrast sensitivity, or tear osmolarity were found among the three groups.

**Table 5.** Usefulness of Physician-Measured Ocular Surface and Tear Film Parameters to Distinguish Presence of Sjogren's Disease in a Clinic-Based Sample of Patients With Dry Eye

					*P* Value	
Parameter/Measure	SDDE (*n* = 20)	Non-SDDE (*n* = 47)	Controls (*n* = 29)	All (*n* = 96)	SDDE vs. Non-SDDE	All
Visual acuity, logMAR units						
Mean	−0.1	−0.1	−0.1	−0.1		
Standard deviation	0.1	0.1	0.1	0.1		
Median	−0.1	−0.1	−0.1	−0.1	0.92	0.87
Interquartile range	[−0.2 to 0]	[−0.2 to 0]	[−0.2 to −0.1]	[−0.2 to 0]		
Range	[−0.3 to 0.1]	[−0.3 to 0.2]	[−0.3 to 0.5]	[−0.3 to 0.5]		
Mars Letter Contrast Sensitivity, log units						
Mean	2	2	2	2		
Standard deviation	0	0	0	0		
Median	2	2	2	2	1.0	1.0
Interquartile range	[2 to 2]	[2 to 2]	[2 to 2]	[2 to 2]		
Range	[2 to 2]	[2 to 2]	[2 to 2]	[2 to 2]		
Tear osmolarity, mOsm/L						
Mean	305	302	303	305		
Standard deviation	12	10	10	11		
Median	302	230	304	302	0.45	0.74
Interquartile range	[298 to 313]	[296 to 309]	[297 to 310]	[296 to 313]		
Range	[287 to 330]	[285 to 334]	[279 to 323]	[279 to 334]		
Schirmer's test without anesthesia, mm/5 mins						
Mean	11	13	18	14		
Standard deviation	9	9	10	9		
Median	8.5	11	17	11	0.15	**0.01**
Interquartile range	[5 to 14]	[7 to 18]	[8 to 27]	[7 to 19]		
Range	[2 to 35]	[4 to 35]	[5 to 35]	[2 to 35]		
TBUT, s						
Mean	3	4	7	5		
Standard deviation	2	2	3	2		
Median	4	4	7	3	0.28	**0.0001**
Interquartile range	[2 to 5]	[3 to 5]	[5 to 8]	[4 to 6]		
Range	[0 to 7]	[0 to 9]	[0 to 11]	[0 to 11]		
Ocular Surface Staining Score – SICCA, total						
Mean	7	4	1	4		
Standard deviation	3	2	1	3		
Median	8	3	1	3	**0.0002**	**0.0001**
Interquartile range	[4 to 9]	[1 to 5]	[0 to 2]	[1 to 6]		
Range	[0 to 11]	[0 to 10]	[0 to 4]	[0 to 11]		
Ocular Surface Staining Score – SICCA, cornea						
Mean	3	2	1	2		
Standard deviation	2	1	1	1		
Median	2.5	1	1	1	**0.0056**	**0.0001**
Interquartile range	[1 to 4]	[1 to 2]	[0 to 2]	[1 to 2]		
Range	[1 to 5]	[0 to 4]	[0 to 1]	[0 to 5]		
Ocular Surface Staining Score – SICCA, conjunctiva						
Mean	4	2	1	2		
Standard deviation	2	2	1	2		
Median	4	2	1	2	**0.0003**	**0.0001**
Interquartile range	[3 to 6]	[1 to 3]	[0 to 1]	[1 to 3]		
Range	[0 to 6]	[0 to 6]	[0 to 3]	[0 to 6]		
Ocular Surface Staining Score – NEI, total						
Mean	16	9	3	9		
Standard deviation	7	6	3	7		
Median	19	7	3	7	**0.0001**	**0.0001**
Interquartile range	[11 to 23]	[3 to 13]	[1 to 4]	[3 to 13]		
Range	[3 to 25]	[0 to 25]	[0 to 11]	[0 to 25]		
Ocular Surface Staining Score – NEI, cornea						
Mean	5	3	1	3		
Standard deviation	3	2	1	3		
Median	5	2	1	2	**0.01**	**0.0001**
Interquartile range	[2 to 8]	[1 to 5]	[0 to 1]	[1 to 4]		
Range	[1 to 11]	[0 to 10]	[0 to 3]	[0 to 11]		
Ocular Surface Staining Score – NEI, conjunctiva						
Mean	11	6	2	6		
Standard deviation	5	5	3	5		
Median	12	6	2	4	**0.0001**	**0.0001**
Interquartile range	[9 to 15]	[2 to 8]	[0 to 3]	[2 to 9]		
Range	[2 to 18]	[0 to 17]	[0 to 9]	[0 to 18]		

NEI, National Eye Institute; SICCA, Sjögren's International Collaborative Clinical Alliance; SSDE, Sjogren's disease related dry eye.

Only right eye values for Tear Osmolarity, Schirmer's Test, TBUT, and Ocular Surface Staining Score (SICCA and NEI) are reported in this table.

Statistically significant *P* values for Kruskal-Wallis test for medians are in bold.

## Discussion

This observational study indicates that among the several commonly used and established tools to measure patient-reported systemic symptoms, only the PROFAD questionnaire demonstrated significantly different median scores between participants with SDDE versus non-SDDE. Upon further analysis of each question, dry mouth-related items, such as experiencing “difficulties in eating,” seemed the most significant difference. The Sjogren's Foundation-recommended short review of systems also showed that the presence of dry mouth was significantly associated with SDDE. There were no statistically significant differences between participants with SDDE versus non-SDDE with regard to ocular symptom scores using multiple questionnaires. One simple, yet effective, way to check for the severity of dry mouth, even in a fast-paced ophthalmology setting, is to ask the patient, “Can you eat a cracker without drinking a fluid or liquid?” An inability to do so significantly increases the likelihood of underlying Sjogren's disease.^37^ Additionally, there were no statistically significant differences between participants with SDDE versus non-SDDE with regard to ocular symptom scores using multiple questionnaires. Both corneal and conjunctival staining scores measured using the NEI as well as the SICCA grading systems were, however, significantly greater among participants with SDDE than participants with non-SDDE. Prior studies have also demonstrated similar findings where patients with SDDE tended to have worse ocular surface staining but similar or even fewer symptoms than patients with non-SDDE.^15^ Whereas improving patient symptoms is an important goal when managing patients with dry eye disease, assessing the ocular surface using vital dye testing irrespective of the severity of patient symptoms should be a priority to ascertain the real extent of surface damage and raise suspicion of underlying Sjogren's disease.^36^ Regrettably, lissamine green staining of the conjunctiva is used much less frequently compared to fluorescein staining of the cornea in evaluating dry eye and ocular surface disease, particularly in Europe, which may lead to underdiagnosis of Sjogren's disese.^38^^,^^39^

The main strength of this study is that it tested several Sjogren's disease symptom assessment tools and detailed ocular surface and tear film parameters concurrently, in a clinic-based sample of patients with dry eye and healthy controls. In addition, patients were assigned into the three study groups according to strict diagnostic criteria for the presence of Sjogren's and dry eye disease. Participants in the non-SSDE had both a negative biopsy and negative serology, eliminating the possibility of misclassification in patients having false negative serology. Our study also has several limitations. The overall number of participants was small, with the number of patients with SDDE being significantly smaller than the non-SSDE and healthy control participants. This unfortunately limits the statistical power of determining differences between SDDE and non-SDDE participants. Additionally, we did not conduct an a priori sample size or power calculation. Rather, we enrolled patients sequentially. This may have prevented certain trending indicators from achieving statistical significance. Regrettably, our study did not inquire about patients’ daily activities requiring sustained gazing, such as reading, driving, or electronic screen usage. Activities that require sustained gazing may lead to worsening of the dry eye findings and visual fatigue.^40^ Although our findings for visual fatigue were nonsignificant, variations in time spent gazing could serve as a possible confounder for dry eye symptoms and signs. Whereas the study did reveal that presence of dry mouth symptoms and worse ocular surface, staining scores are likely indicators of an underling Sjogren's disease in patients with dry eye disease, we did not establish explicit thresholds for these parameters. Last, we did not adjust for systemic treatment status in Sjogren's disease participants. Although systemic treatments are effective in treating extraglandular symptoms, prior work has generally shown that symptoms of dryness and fatigue or joint pains are not generally not targeted nor adequately improved with systemic treatment.^41^^,^^42^ Thus, we do not anticipate adjusting for systemic treatment use would have an impact on our results. Finally, we did not randomize the order of administering the questionnaires, which could have potentially influenced our results due to fatigue in answering questionnaires at the end.

## Conclusions

This study provides evidence that ocular surface vital dye staining must be performed in all patients presenting with dry eye symptoms, irrespective of the severity of their ocular or systemic symptoms; a critical step toward identifying patients who would benefit from receiving Sjogren's disease evaluations. In this era of several targeted biological treatments being developed for treating Sjogren's disease, accurate timely diagnosis and early treatment may ultimately minimize long-term ocular and systemic morbidity and reduce the associated personal and societal burden and costs.

## Supplementary Material

Supplement 1

## References

[1] Stapleton F, Alves M, Bunya VY, et al. TFOS DEWS II epidemiology report. *Ocul Surf*. 2017; 15(3): 334–365. 28736337 10.1016/j.jtos.2017.05.003

[2] Dana R, Bradley JL, Guerin A, et al. Estimated prevalence and incidence of dry eye disease based on coding analysis of a large, all-age United States health care system. *Am J Ophthalmol*. 2019; 202: 47–54. 30721689 10.1016/j.ajo.2019.01.026

[3] McCann P, Abraham AG, Mukhopadhyay A, et al. Prevalence and incidence of dry eye and meibomian gland dysfunction in the United States: a systematic review and meta-analysis. *JAMA Ophthalmol*. 2022; 140(12): 1181–1192. 36301551 10.1001/jamaophthalmol.2022.4394 PMC9614673

[4] Karakus S, Mathews PM, Agrawal D, Henrich C, Ramulu PY, Akpek EK. Impact of dry eye on prolonged reading. *Optom Vis Sci Off Publ Am Acad Optom*. 2018; 95(12): 1105–1113. 10.1097/OPX.0000000000001303 30439719

[5] Uchino M, Schaumberg DA. Dry eye disease: impact on quality of life and vision. *Curr Ophthalmol Rep*. 2013; 1(2): 51–57. 23710423 10.1007/s40135-013-0009-1 PMC3660735

[6] Saldanha IJ, Bunya VY, McCoy SS, Makara M, Baer AN, Akpek EK. Ocular manifestations and burden related to Sjögren syndrome: results of a patient survey. *Am J Ophthalmol*. 2020; 219: 40–48. 32569739 10.1016/j.ajo.2020.05.043 PMC7606749

[7] Xiong F, Pula D, Akpek EK, et al. Sjögren's versus non-Sjögren's ocular features: similar symptoms, but significantly worse signs. *Invest Ophthalmol Vis Sci*. 2024; 65(1): 23. 10.1167/iovs.65.1.23 PMC10784845 38193760

[8] Cui D, Mathews P, Li G, VanCourt S, Akpek E. Outcomes of Sjögren's versus non-Sjögren's related dry eye in a longitudinal, tertiary clinic-based sample. *PLoS One*. 2021; 16(12): e0261241. 34919587 10.1371/journal.pone.0261241 PMC8682907

[9] Mathews PM, Hahn S, Hessen M, et al. Ocular complications of primary Sjögren syndrome in men. *Am J Ophthalmol*. 2015; 160(3): 447–452. e1. 26093285 10.1016/j.ajo.2015.06.004

[10] Singh S, Das AV, Basu S. Ocular involvement in Sjögren syndrome: risk factors for severe visual impairment and vision-threatening corneal complications. *Am J Ophthalmol*. 2021; 225: 11–17. 33385368 10.1016/j.ajo.2020.12.019

[11] Akpek EK, Mathews P, Hahn S, et al. Ocular and systemic morbidity in a longitudinal cohort of Sjögren's syndrome. *Ophthalmology*. 2015; 122(1): 56–61. 25178806 10.1016/j.ophtha.2014.07.026

[12] Kassan SS, Moutsopoulos HM. Clinical manifestations and early diagnosis of Sjögren syndrome. *Arch Intern Med*. 2004; 164(12): 1275–1284. 15226160 10.1001/archinte.164.12.1275

[13] Nocturne G, Pontarini E, Bombardieri M, Mariette X. Lymphomas complicating primary Sjögren's syndrome: from autoimmunity to lymphoma. *Rheumatol Oxf Engl*. 2021; 60(8): 3513–3521. 10.1093/rheumatology/kez052 PMC8328496 30838413

[14] Akpek EK, Lindsley KB, Adyanthaya RS, Swamy R, Baer AN, McDonnell PJ. Treatment of Sjögren's syndrome-associated dry eye an evidence-based review. *Ophthalmology*. 2011; 118(7): 1242–1252. 21459453 10.1016/j.ophtha.2010.12.016

[15] Akpek EK, Bunya VY, Saldanha IJ. Sjögren's syndrome: more than just dry eye. *Cornea*. 2019; 38(5): 658–661. 30681523 10.1097/ICO.0000000000001865 PMC6482458

[16] Liew MSH, Zhang M, Kim E, Akpek EK. Prevalence and predictors of Sjogren's syndrome in a prospective cohort of patients with aqueous-deficient dry eye. *Br J Ophthalmol*. 2012; 96(12): 1498–1503. 23001257 10.1136/bjophthalmol-2012-301767

[17] Yu K, Bunya V, Maguire M, Asbell P, Ying GS, Dry Eye Assessment and Management Study Research Group. Systemic conditions associated with severity of dry eye signs and symptoms in the Dry Eye Assessment and Management Study. *Ophthalmology*. 2021; 128(10): 1384–1392. 33785415 10.1016/j.ophtha.2021.03.030 PMC8463420

[18] Bunya VY, Fernandez KB, Ying GS, et al. Survey of ophthalmologists regarding practice patterns for dry eye and Sjogren syndrome. *Eye Contact Lens*. 2018; 44 Suppl 2(Suppl 2): S196–S201. 29369232 10.1097/ICL.0000000000000448 PMC6046269

[19] Akpek EK, Klimava A, Thorne JE, Martin D, Lekhanont K, Ostrovsky A. Evaluation of patients with dry eye for presence of underlying Sjögren syndrome. *Cornea*. 2009; 28(5): 493–497. doi:10.1097/ICO.0b013e31818d3846. 19421051 PMC2693267

[20] Komori K, Komori M, Horino T, Nishiyama S, Takei M, Suganuma N. Factors associated with delayed diagnosis of Sjögren's syndrome among members of the Japanese Sjögren's Association for Patients. *Clin Exp Rheumatol*. 2021; 39 Suppl 133(6): 146–152. 10.55563/clinexprheumatol/s8l2n0 33822710

[21] Huang YT, Lu TH, Chou PL, Weng MY. Diagnostic delay in patients with primary Sjögren's syndrome: a population-based cohort study in Taiwan. *Healthc Basel Switz*. 2021; 9(3): 363. 10.3390/healthcare9030363 PMC8004927 33807070

[22] Shiboski CH, Shiboski SC, Seror R, et al. 2016 American College of Rheumatology/European League Against Rheumatism Classification Criteria for Primary Sjögren's Syndrome: a consensus and data-driven methodology involving three international patient cohorts. *Arthritis Rheumatol Hoboken NJ*. 2017; 69(1): 35–45. 10.1002/art.39859 PMC5650478 27785888

[23] Salvi J . Columbia-Suicide Severity Rating Scale (C-SSRS). *Emerg Med Pract*. 2019; 21(5): CD3–CD4. PMC7974826 31039299

[24] Wang YP, Gorenstein C. Psychometric properties of the Beck Depression Inventory-II: a comprehensive review. *Rev Bras Psiquiatr Sao Paulo Braz 1999*. 2013; 35(4): 416–431. 10.1590/1516-4446-2012-1048 24402217

[25] Tombaugh TN, McIntyre NJ. The mini-mental state examination: a comprehensive review. *J Am Geriatr Soc.* 1992; 40(9): 922–935. doi:10.1111/j.1532-5415.1992.tb01992.x. 1512391

[26] Park SH, Yu HY. How useful is the center for epidemiologic studies depression scale in screening for depression in adults? An updated systematic review and meta-analysis. *Psychiatry Res*. 2021; 302: 114037. 34098160 10.1016/j.psychres.2021.114037

[27] Lins L, Carvalho FM. SF-36 total score as a single measure of health-related quality of life: scoping review. *SAGE Open Med*. 2016; 4: 2050312116671725. 27757230 10.1177/2050312116671725 PMC5052926

[28] Raymond K, Maher S, Saucier CD, et al. Validation of the PROFAD-SSI-SF in patients with primary Sjögren's syndrome with organ involvement: results of qualitative interviews and psychometric analyses. *Rheumatol Ther*. 2022; 10(1): 95–115. 36227531 10.1007/s40744-022-00493-2 PMC9931977

[29] Ramos-Casals M, Brito-Zerón P, Bombardieri S, et al. EULAR recommendations for the management of Sjögren's syndrome with topical and systemic therapies. *Ann Rheum Dis*. 2020; 79(1): 3–18. 31672775 10.1136/annrheumdis-2019-216114

[30] Sjögren's Foundation. Sjögren's Foundation. April 15, 2025. Accessed May 12, 2025. Available at: https://sjogrens.org/home.

[31] Schiffman RM, Christianson MD, Jacobsen G, Hirsch JD, Reis BL. Reliability and validity of the Ocular Surface Disease Index. *Arch Ophthalmol Chic Ill 1960*. 2000; 118(5): 615–621. 10.1001/archopht.118.5.615 10815152

[32] Abetz L, Rajagopalan K, Mertzanis P, et al. Development and validation of the Impact of Dry Eye on Everyday Life (IDEEL) questionnaire, a patient-reported outcomes (PRO) measure for the assessment of the burden of dry eye on patients. *Health Qual Life Outcomes*. 2011; 9: 111. 22152125 10.1186/1477-7525-9-111 PMC3269387

[33] Mangione CM, Lee PP, Gutierrez PR, et al. Development of the 25-item National Eye Institute Visual Function Questionnaire. *Arch Ophthalmol Chic Ill 1960*. 2001; 119(7): 1050–1058. 10.1001/archopht.119.7.1050 11448327

[34] Schaumberg DA, Gulati A, Mathers WD, et al. Development and validation of a short global dry eye symptom index. *Ocul Surf*. 2007; 5(1): 50–57. 17252166 10.1016/s1542-0124(12)70053-8

[35] Whitcher JP, Shiboski CH, Shiboski SC, et al. A simplified quantitative method for assessing keratoconjunctivitis sicca from the Sjögren's Syndrome International Registry. *Am J Ophthalmol*. 2010; 149(3): 405–415. 20035924 10.1016/j.ajo.2009.09.013 PMC3459675

[36] Lemp MA . Report of the National Eye Institute/Industry workshop on Clinical Trials in Dry Eyes. *CLAO J Off Publ Contact Lens Assoc Ophthalmol Inc*. 1995; 21(4): 221–232. 8565190

[37] Bunya VY, Maguire MG, Akpek EK, et al. A new screening questionnaire to identify patients with dry eye with a high likelihood of having Sjögren syndrome. *Cornea*. 2021; 40(2): 179–187. 33055548 10.1097/ICO.0000000000002515 PMC7779700

[38] Sall K, Foulks GN, Pucker AD, Ice KL, Zink RC, Magrath G. Validation of a modified National Eye Institute grading scale for corneal fluorescein staining. *Clin Ophthalmol Auckl NZ*. 2023; 17: 757–767. 10.2147/OPTH.S398843 PMC10007867 36915716

[39] Cartes C, Segovia C, Calonge M, Figueiredo FC. International survey on dry eye diagnosis by experts. *Heliyon*. 2023; 9(6): e16995. 37484334 10.1016/j.heliyon.2023.e16995 PMC10361019

[40] Akpek EK, Karakus S, Ramulu PY, Mathews PM. Sustained gazing causes measurable decline in visual function of patients with dry eye. *Am J Ophthalmol*. 2020; 210: 107–115. 31606440 10.1016/j.ajo.2019.10.009

[41] Wang X, Zhang T, Guo Z, et al. The efficiency of hydroxychloroquine for the treatment of primary Sjögren's syndrome: a systematic review and meta-analysis. *Front Pharmacol*. 2021; 12: 693796. 34588979 10.3389/fphar.2021.693796 PMC8475756

[42] Brito-Zerón P, Retamozo S, Kostov B, et al. Efficacy and safety of topical and systemic medications: a systematic literature review informing the EULAR recommendations for the management of Sjögren's syndrome. *RMD Open*. 2019; 5(2): e001064. 31749986 10.1136/rmdopen-2019-001064 PMC6827762

