# Supplementary material for: Patient-Reported Symptoms Versus Clinician-Measured Signs to Distinguish Sjogren's in Patients With Dry Eye
Source: Transl Vis Sci Technol. 2026 Jan 22;15(1):27. doi: 10.1167/tvst.15.1.27 (PMC12849820; doi:10.1167/tvst.15.1.27)
Supplement: Supplement 1 [file tvst-15-1-27_s001.zip › Appendix A PROFAD.pdf]

PROFILE OF FATIGUE AND DISCOMFORT
Short Form (19 question items)

Please assess how bad at worst each symptom has been in the last two weeks by ringing one of the numbers 0 to 7.

1. The **worst** problem that I've had in the last two weeks with **needing to rest,** feeling **tired,** being **exhausted or needing to sleep:**

   no need to rest at all 0 1 2 3 4 5 6 7 as bad as imaginable

2. The **worst** problem that I've had in the last two weeks with it **being hard to GET going, things taking an effort** or me feeling that **'it's a battle':**

   not hard to get going at all 0 1 2 3 4 5 6 7 as bad as imaginable

3. The **worst** problem that I've had in the last two weeks with it **being hard to KEEP going,** me being **easily worn out** or **lacking in energy:**

   not hard to keep going at all 0 1 2 3 4 5 6 7 as bad as imaginable

4. The **worst** problem that I've had in the last two weeks with **lack of strength in my muscles** or **feeling weak:**

   no lack of strength at all 0 1 2 3 4 5 6 7 as bad as imaginable

5. The **worst** problem I've had in the last two weeks with **not thinking clearly** or finding it **hard to concentrate:**

   no such problem at all 0 1 2 3 4 5 6 7 as bad as imaginable

6. The **worst** problem I've had in the last two weeks with **forgetting things** or **making mistakes:**

   no such problem at all 0 1 2 3 4 5 6 7 as bad as imaginable

[Questions continue on next page]

7. The **worst** problem I've had in the last two weeks with **discomfort in my limbs:**
e.g. discomfort, aches or pains in your big joints (hips, knees, shoulders) or in your muscles or aching all over

no problem at all 0 1 2 3 4 5 6 7 as bad as imaginable

8. **Worst** problem in the last 2 weeks with **discomfort or swelling of fingers or wrists:**

no problem at all 0 1 2 3 4 5 6 7 as bad as imaginable

9. The **worst** problem I've had in the last two weeks with **uncomfortably cold hands:**

no problem at all 0 1 2 3 4 5 6 7 as bad as imaginable

10. The **worst** problem that I've had in the last two weeks with **dry or itchy skin:**

no problem at all 0 1 2 3 4 5 6 7 as bad as imaginable

11. The **worst** problem that I've had in the last two weeks with **vaginal dryness:**
e.g. experienced discomfort during sex due to vaginal dryness

no problem at all 0 1 2 3 4 5 6 7 as bad as imaginable

12. The **worst** problem that I've had in the last two weeks with **sore eyes:**
e.g. eyes felt gritty, painful eyes, burning eyes, itchy eyes or irritation in eyes

no problem at all 0 1 2 3 4 5 6 7 as bad as imaginable

13. The **worst** problem that I've had in the last two weeks with **eye irritation:**
e.g. eyes irritated by smoky atmosphere, eyes were uncomfortable in the wind, eyes were uncomfortable in air-conditioning or low-humidity places

no problem at all 0 1 2 3 4 5 6 7 as bad as imaginable

[Questions continue on next page]

14. The **worst** problem that I've had in the last two weeks with **poor vision:**
(even if wearing spectacles) e.g., blurred vision, poor vision, problem with eyes limited reading, watching TV or night driving, hard to see computer screen or cash machine screen

no problem at all 0 1 2 3 4 5 6 7 as bad as imaginable

15. The **worst** problem that I've had in the last two weeks with **difficulties in eating:**
e.g. mouth felt dry when eating, difficult to swallow foods, needed liquid to swallow food, food stuck to the mouth, needed to rinse away remains of food or have appreciated food less

no problem at all 0 1 2 3 4 5 6 7 as bad as imaginable

16. The **worst** problem that I've had in the last two weeks with **dry throat or nose:**
e.g. mouth felt dry when breathing, had difficulty talking with dry mouth, needed a drink to talk easily, nose felt dry, dry throat, air-conditioning dried mouth

no problem at all 0 1 2 3 4 5 6 7 as bad as imaginable

17. The **worst** problem that I've had in the last two weeks with **bad breath:**
e.g. felt that your breath has smelt, saliva felt sticky

no problem at all 0 1 2 3 4 5 6 7 as bad as imaginable

18. The **worst** problem in the last two weeks with **needing fluid to wet my mouth:**
e.g. carried drink to bed, needed drink during the night, woke at night to pass urine, had urgent need to pass urine

no problem at all 0 1 2 3 4 5 6 7 as bad as imaginable

19. The **worst** problem that I've had in the last two weeks with **other mouth problems:**
e.g. mouth ulcers, swollen salivary glands, felt as though choking because of dryness, change in flavours or tastes, needed to visit the dentists

no problem at all 0 1 2 3 4 5 6 7 as bad as imaginable

Thank you for answering these questions.

© D.A. Booth & S.J. Bowman, April 2002
