# Supplementary material for: Patient-Reported Symptoms Versus Clinician-Measured Signs to Distinguish Sjogren's in Patients With Dry Eye
Source: Transl Vis Sci Technol. 2026 Jan 22;15(1):27. doi: 10.1167/tvst.15.1.27 (PMC12849820; doi:10.1167/tvst.15.1.27)
Supplement: Supplement 1 [file tvst-15-1-27_s001.zip › Appendix A SF36.pdf]

Medical Outcomes Study Questionnaire Short Form 36 Health Survey (SF-36)

About: The SF-36 is an indicator of overall health status.

Items: 10

Reliability: Most of these studies that examined the reliability of the SF_36 have exceeded 0.80 (McHorney et al., 1994; Ware et al., 1993). Estimates of reliability in the physical and mental sections are typically above 0.90.

Validity: The SF-36 is also well validated.

Scoring:
The SF-36 has eight scaled scores; the scores are weighted sums of the questions in each section. Scores range from 0 - 100
Lower scores = more disability, higher scores = less disability
Sections:

- Vitality
- Physical functioning
- Bodily pain
- General health perceptions
- Physical role functioning
- Emotional role functioning
- Social role functioning
- Mental health

References:

McHorney CA, Ware JE, Lu JFR, Sherbourne CD. The MOS 36-Item Short-Form Health Survey (SF-36®): III. tests of data quality, scaling assumptions and reliability across diverse patient groups. *Med Care*1994; 32(4):40-66.

Ware JE, Snow KK, Kosinski M, Gandek B. *SF-36® Health Survey Manual and Interpretation Guide*. Boston, MA: New England Medical Center, The Health Institute, 1993.

Ware JE, Sherbourne CD. The MOS 36-Item Short-Form Health Survey (SF-36®): I. conceptual framework and item selection. Med Care 1992; 30(6):473-83.

Medical Outcomes Study Questionnaire Short Form 36 Health Survey

This survey asks for your views about your health. This information will help keep track of how you feel and how well you are able to do your usual activities. Thank you for completing this survey! For each of the following questions, please circle the number that best describes your answer.

**1. In general, would you say your health is:**	
Excellent	1
Very good	2
Good	3
Fair	4
Poor	5
**2. Compared to one year ago,**	
Much better now than one year ago	1
Somewhat better now than one year ago	2
About the same	3
Somewhat worse now than one year ago	4
Much worse now than one year ago	5

3. The following items are about activities you might do during a typical day. Does your health now limit you in these activities? If so, how much?
(Circle One Number on Each Line)

	**Yes, Limited a Lot (1)**	**Yes, Limited a Little (2)**	**No, Not limited at All (3)**
a. **Vigorous activities**, such as running, lifting heavy objects, participating in strenuous sports	1	2	3
b. **Moderate activities**, such as moving a table, pushing a vacuum cleaner, bowling, or playing golf	1	2	3
c. Lifting or carrying groceries	1	2	3
d. Climbing **several** flights of stairs	1	2	3
e. Climbing **one** flight of stairs	1	2	3
f. Bending, kneeling, or stooping	1	2	3

g. Walking **more than a mile**	1	2	3
h. Walking **several blocks**	1	2	3
i. Walking **one block**	1	2	3
j. Bathing or dressing yourself	1	2	3

4. During the **past 4 weeks**, have you had any of the following problems with your work or other regular daily activities **as a result of your physical health**?
(Circle One Number on Each Line)

	**Yes (1)**	**No (2)**
a. Cut down the amount of time you spent on work or other activities	1	2
b. **Accomplished less** than you would like	1	2
c. Were limited in the **kind** of work or other activities	1	2
d. Had **difficulty** performing the work or other activities (for example, it took extra effort)	1	2

5. During the **past 4 weeks,** have you had any of the following problems with your work or other regular daily activities **as a result of any emotional problems** (such as feeling depressed or anxious)?
(Circle One Number on Each Line)

	**Yes**	**No**
a. Cut down the amount of time you spent on work or other activities	1	2
b. **Accomplished less** than you would like	1	2
c. Didn't do work or other activities as **carefully** as usual	1	2

**6. During the past 4 weeks, to what extent has your physical health or emotional problems interfered with your normal social activities with family, friends, neighbors, or groups?**	
Not at all	1
Slightly	2
Moderately	3
Quite a bit	4
Extremely	5

**7. How much bodily pain have you had during the past 4 weeks?**	
None	1
Very mild	2
Mild	3
Moderate	4
Severe	5
Very severe	6
**8. During the past 4 weeks, how much did pain interfere with your normal work (including both work outside the home and housework)?**	
Not at all	1
A little bit	2
Moderately	3
Quite a bit	4
Extremely	5

These questions are about how you feel and how things have been with you **during the past 4 weeks**. For each question, please give the one answer that comes closest to the way you have been feeling. **(Circle One Number on Each Line)**

9. How much of the time during the **past 4 weeks** . . .

	**All of the Time**	**Most of the Time**	**A Good Bit of the Time**	**Some of the Time**	**A Little of the Time**	**None of the Time**
a. Did you feel full of pep?	1	2	3	4	5	6
b. Have you been a very nervous person?	1	2	3	4	5	6
c. Have you felt so down in the dumps that nothing could cheer you up?	1	2	3	4	5	6
d. Have you felt calm and peaceful?	1	2	3	4	5	6
e. Did you have a lot of energy?	1	2	3	4	5	6

	All of the Time	Most of the Time	A Good Bit of the Time	Some of the Time	A Little of the Time	None of the Time
f. Have you felt downhearted and blue?	1	2	3	4	5	6
g. Did you feel worn out?	1	2	3	4	5	6
h. Have you been a happy person?	1	2	3	4	5	6
i. Did you feel tired?	1	2	3	4	5	6

**10. During the past 4 weeks, how much of the time has your physical health or emotional problems interfered with your social activities (like visiting with friends, relatives, etc.)? (Circle One Number)**	
All of the time	1
Most of the time	2
Some of the time	3
A little of the time	4
None of the time	5

11. How TRUE or FALSE is each of the following statements for you. (Circle One Number on Each Line)

	Definitely True	Mostly True	Don't Know	Mostly False	Definitely False
a. I seem to get sick a little easier than other people	1	2	3	4	5
b. I am as healthy as anybody I know	1	2	3	4	5
c. I expect my health to get worse	1	2	3	4	5
d. My health is excellent	1	2	3	4	5
