# Supplementary material for: Patient-Reported Symptoms Versus Clinician-Measured Signs to Distinguish Sjogren's in Patients With Dry Eye
Source: Transl Vis Sci Technol. 2026 Jan 22;15(1):27. doi: 10.1167/tvst.15.1.27 (PMC12849820; doi:10.1167/tvst.15.1.27)
Supplement: Supplement 1 [file tvst-15-1-27_s001.zip › Appendix A VDAS.pdf]

Visual Dryness Analogue Scale (VDAS)

This study will use a Visual Analogue Scale scoring system on a 0-100 point scale to evaluate eye dryness.

Subjects will be asked to complete the following VAS regarding their **current** eye dryness.

Subjects are asked to rate their eye dryness in both eyes by placing a vertical mark on the horizontal line on the paper scale to indicate the level of discomfort.

Zero corresponds to “no discomfort” and 100 correspond to “maximal discomfort.”

The subject must sign and date the bottom of the page to acknowledge completion of the assessment.

0 50 100

“no discomfort” “maximum discomfort”

VDA: _____ mm
