# Supplementary material for: Patient-Reported Symptoms Versus Clinician-Measured Signs to Distinguish Sjogren's in Patients With Dry Eye
Source: Transl Vis Sci Technol. 2026 Jan 22;15(1):27. doi: 10.1167/tvst.15.1.27 (PMC12849820; doi:10.1167/tvst.15.1.27)
Supplement: Supplement 1 [file tvst-15-1-27_s001.zip › Appendix A VFAS.pdf]

Visual Fatigue Analogue Scale (VFAS)

This study will use a Visual Fatigue Scale scoring system on a 0-100 point scale to evaluate eye fatigue.

Subjects will be asked to complete the following VAS regarding their **current** eye fatigue.

Subjects will be reminded this is correlated to ocular fatigue and not physical fatigue.

Subjects are asked to rate their eye fatigue in both eyes by placing a vertical mark on the horizontal line on the paper scale to indicate the level of tiredness.

Zero corresponds to "not tired at all" and 100 correspond to "eyes are very tired."

The subject must sign and date the bottom of the page to acknowledge completion of the assessment.

0 50 100

"eyes not tired at all" "eyes very tired"

VFA: _____ mm
