# Supplementary material for: Patient-Reported Symptoms Versus Clinician-Measured Signs to Distinguish Sjogren's in Patients With Dry Eye
Source: Transl Vis Sci Technol. 2026 Jan 22;15(1):27. doi: 10.1167/tvst.15.1.27 (PMC12849820; doi:10.1167/tvst.15.1.27)
Supplement: Supplement 1 [file tvst-15-1-27_s001.zip › Appendix A VFQ25.pdf]

PB/SA

National Eye Institute
Visual Functioning Questionnaire - 25
(VFQ-25)

version 2000

(SELF-ADMINISTERED FORMAT)

January 2000

RAND hereby grants permission to use the "National Eye Institute Visual Functioning Questionnaire 25 (VFQ-25) July 1996, in accordance with the following conditions which shall be assumed by all to have been agreed to as a consequence of accepting and using this document:

1. Changes to the NEI VFQ-25 - July 1996 may be made without the written permission of RAND. However, all such changes shall be clearly identified as having been made by the recipient.

2. The user of this NEI VFQ-25 - July 1996 accepts full responsibility, and agrees to hold RAND harmless, for the accuracy of any translations of the NEI VFQ-25 Test Version - July 1996 into another language and for any errors, omissions, misinterpretations, or consequences thereof.

3. The user of this NEI VFQ-25 - July 1996 accepts full responsibility, and agrees to hold RAND harmless, for any consequences resulting from the use of the NEI VFQ-25.

4. The user of the NEI VFQ-25 - July 1996 will provide a credit line when printing and distributing this document or in publications of results or analyses based on this instrument acknowledging that it was developed at RAND under the sponsorship of the National Eye Institute.

5. No further written permission is needed for use of this NEI VFQ-25 - July 1996.

7/29/96

© R 1996

The following is a survey with statements about problems which involve your vision or feelings that you have about your vision condition. After each question please choose the response that best describes your situation.

Please answer all the questions as if you were wearing your glasses or contact lenses (if any).

Please take as much time as you need to answer each question. All your answers are confidential. In order for this survey to improve our knowledge about vision problems and how they affect your quality of life, your answers must be as accurate as possible. Remember, if you wear glasses or contact lenses, please answer all of the following questions as though you were wearing them.

INSTRUCTIONS:

1. In general we would like to have people try to complete these forms on their own. If you find that you need assistance, please feel free to ask the project staff and they will assist you.

2. Please answer every question (unless you are asked to skip questions because they don't apply to you).

3. Answer the questions by circling the appropriate number.

4. If you are unsure of how to answer a question, please give the best answer you can and make a comment in the left margin.

5. Please complete the questionnaire before leaving the center and give it to a member of the project staff. Do not take it home.

6. If you have any questions, please feel free to ask a member of the project staff, and they will be glad to help you.

STATEMENT OF CONFIDENTIALITY:

All information that would permit identification of any person who completed this questionnaire will be regarded as strictly confidential. Such information will be used only for the purposes of this study and will not be disclosed or released for any other purposes without prior consent, except as required by law.

© R 1996

Visual Functioning Questionnaire - 25

PART 1 - GENERAL HEALTH AND VISION

1. <u>In general,</u> would you say your overall <u>health</u> is:

(Circle One)

**Excellent**	**1**
**Very Good**	**2**
**Good**	**3**
**Fair**	**4**
**Poor**	**5**

2. At the present time, would you say your eyesight using both eyes (with glasses or contact lenses, if you wear them) is <u>excellent</u>, <u>good</u>, <u>fair</u>, <u>poor</u>, or <u>very</u> <u>poor</u> or are you <u>completely blind</u>?

(Circle One)

**Excellent**	**1**
**Good**	**2**
**Fair**	**3**
**Poor**	**4**
**Very Poor**	**5**
**Completely Blind**	**6**

© R 1996

3. How much of the time do you worry about your eyesight?

(Circle One)

None of the time.......................... 1
A little of the time........................ 2
Some of the time......................... 3
Most of the time 4
All of the time?............................ 5

4. How much pain or discomfort have you had in and around your eyes (for example, burning, itching, or aching)? Would you say it is:

(Circle One)

None 1
Mild.................................... 2
Moderate 3
Severe, or........................... 4
Very severe?...................... 5

PART 2 - DIFFICULTY WITH ACTIVITIES

The next questions are about how much difficulty, if any, you have doing certain activities wearing your glasses or contact lenses if you use them for that activity.

5. How much difficulty do you have reading ordinary print in newspapers? Would you say you have:

(Circle One)

No difficulty at all... 1
A little difficulty.. 2
Moderate difficulty.. 3
Extreme difficulty.. 4
Stopped doing this because of your eyesight.... 5
Stopped doing this for other reasons or not interested in doing this 6

© R 1996

6. How much difficulty do you have doing work or hobbies that require you to <u>see well up close</u>, such as cooking, sewing, fixing things around the house, or using hand tools? Would you say:

(Circle One)

No difficulty at all.. 1

A little difficulty... 2

Moderate difficulty.. 3

Extreme difficulty.. 4

Stopped doing this because of your eyesight.... 5

Stopped doing this for other reasons or not interested in doing this 6

7. Because of your eyesight, how much difficulty do you have <u>finding something on a crowded shelf</u>?

(Circle One)

No difficulty at all.. 1

A little difficulty... 2

Moderate difficulty.. 3

Extreme difficulty.. 4

Stopped doing this because of your eyesight.... 5

Stopped doing this for other reasons or not interested in doing this 6

8. How much difficulty do you have <u>reading street signs or the names of stores</u>?

(Circle One)

No difficulty at all.. 1

A little difficulty... 2

Moderate difficulty.. 3

Extreme difficulty.. 4

Stopped doing this because of your eyesight.... 5

Stopped doing this for other reasons or not interested in doing this 6

© R 1996

9. **Because of your eyesight, how much difficulty do you have <u>going down steps, stairs, or curbs in dim light or at night</u>?**

(Circle One)

No difficulty at all.. 1
A little difficulty.. 2
Moderate difficulty.. 3
Extreme difficulty.. 4
Stopped doing this because of your eyesight.... 5
Stopped doing this for other reasons or not interested in doing this 6

10. **Because of your eyesight, how much difficulty do you have <u>noticing objects off to the side while you are walking along</u>?**

(Circle One)

No difficulty at all.. 1
A little difficulty.. 2
Moderate difficulty.. 3
Extreme difficulty.. 4
Stopped doing this because of your eyesight.... 5
Stopped doing this for other reasons or not interested in doing this 6

11. **Because of your eyesight, how much difficulty do you have <u>seeing how people react to things</u> you say?**

(Circle One)

No difficulty at all.. 1
A little difficulty.. 2
Moderate difficulty.. 3
Extreme difficulty.. 4
Stopped doing this because of your eyesight.... 5
Stopped doing this for other reasons or not interested in doing this 6

© R 1996

12. **Because of your eyesight, how much difficulty do you have <u>picking out and matching your own clothes</u>?**

(Circle One)

No difficulty at all.. 1
A little difficulty... 2
Moderate difficulty.. 3
Extreme difficulty.. 4
Stopped doing this because of your eyesight.... 5
Stopped doing this for other reasons or not interested in doing this 6

13. **Because of your eyesight, how much difficulty do you have <u>visiting with people in their homes, at parties, or in restaurants</u> ?**

(Circle One)

No difficulty at all.. 1
A little difficulty... 2
Moderate difficulty.. 3
Extreme difficulty.. 4
Stopped doing this because of your eyesight.... 5
Stopped doing this for other reasons or not interested in doing this 6

14. **Because of your eyesight, how much difficulty do you have <u>going out to see movies, plays, or sports events</u>?**

(Circle One)

No difficulty at all.. 1
A little difficulty... 2
Moderate difficulty.. 3
Extreme difficulty.. 4
Stopped doing this because of your eyesight.... 5
Stopped doing this for other reasons or not interested in doing this 6

© R 1996

15. Are you currently driving, at least once in a while?

(Circle One)

Yes .... 1 *Skip To Q 15c*

No 2

15a. IF NO: Have you never driven a car or have you given up driving?

(Circle One)

Never drove ...... 1 *Skip To Part 3, Q 17*

Gave up............. 2

15b. IF YOU GAVE UP DRIVING: Was that mainly because of your eyesight, mainly for some other reason, or because of both your eyesight and other reasons?

(Circle One)

Mainly eyesight .... 1 *Skip To Part 3, Q 17*

Mainly other reasons .... 2 *Skip To Part 3, Q 17*

Both eyesight and other reasons ... 3 *Skip To Part 3, Q 17*

15c. IF CURRENTLY DRIVING: How much difficulty do you have driving during the daytime in familiar places? Would you say you have:

(Circle One)

No difficulty at all 1
A little difficulty 2
Moderate difficulty 3
Extreme difficulty 4

© R 1996

16. How much difficulty do you have <u>driving at night</u>? Would you say you have:

(Circle One)

No difficulty at all.................................. 1

A little difficulty..................................... 2

Moderate difficulty................................ 3

Extreme difficulty.................................. 4

Have you stopped doing this because of your eyesight................................ 5

Have you stopped doing this for other reasons or are you not interested in doing this 6

16A. How much difficulty do you have <u>driving in difficult conditions, such as in bad weather, during rush hour, on the freeway, or in city traffic</u>? Would you say you have:

(Circle One)

No difficulty at all.................................. 1

A little difficulty..................................... 2

Moderate difficulty................................ 3

Extreme difficulty.................................. 4

Have you stopped doing this because of your eyesight................................ 5

Have you stopped doing this for other reasons or are you not interested in doing this 6

© R 1996

PART 3: RESPONSES TO VISION PROBLEMS

The next questions are about how things you do may be affected by your vision. For each one, please circle the number to indicate whether for you the statement is true for you all, most, some, a little, or none of the time.

(Circle One On Each Line)

READ CATEGORIES:	All of the time	Most of the time	Some of the time	A little of the time	None of the time
17. Do you accomplish less than you would like because of your vision?	1	2	3	4	5
18. Are you limited in how long you can work or do other activities because of your vision?	1	2	3	4	5
19. How much does pain or discomfort in or around your eyes, for example, burning, itching, or aching, keep you from doing what you'd like to be doing? Would you say:	1	2	3	4	5

© R 1996

For each of the following statements, please circle the number to indicate whether for you the statement is definitely true, mostly true, mostly false, or definitely false for you or you are not sure.

(Circle One On Each Line)

	Definitely True	Mostly True	Not Sure	Mostly False	Definitely False
**20.** I stay home most of the time because of my eyesight.....	1	2	3	4	5
**21.** I feel frustrated a lot of the time because of my eyesight..............................	1	2	3	4	5
**22.** I have much less control over what I do, because of my eyesight.	1	2	3	4	5
**23.** Because of my eyesight, I have to rely too much on what other people tell me. .	1	2	3	4	5
**24.** I need a lot of help from others because of my eyesight..............................	1	2	3	4	5
**25.** I worry about doing things that will embarrass myself or others, because of my eyesight..............................	1	2	3	4	5

© R 1996

Appendix of Optional Additional Questions

SUBSCALE: GENERAL HEALTH

A1. How would you rate your overall health, on a scale where zero is as bad as death and 10 is best possible health?

(Circle One)

0	1	2	3	4	5	6	7	8	9	10
Worst										Best

SUBSCALE: GENERAL VISION

A2. How would you rate your eyesight now (with glasses or contact lens on, if you wear them), on a scale of from 0 to 10, where zero means the worst possible eyesight, as bad or worse than being blind, and 10 means the best possible eyesight?

(Circle One)

0	1	2	3	4	5	6	7	8	9	10
Worst										Best

SUBSCALE: NEAR VISION

A3. Wearing glasses, how much difficulty do you have reading the small print in a telephone book, on a medicine bottle, or on legal forms? Would you say:

(Circle One)

No difficulty at all	1
A little difficulty	2
Moderate difficulty	3
Extreme difficulty	4
Stopped doing this because of your eyesight	5
Stopped doing this for other reasons or not interested in doing this	6

© R 1996

A4. Because of your eyesight, how much difficulty do you have figuring out whether bills you receive are accurate?

(Circle One)

No difficulty at all.. 1
A little difficulty... 2
Moderate difficulty.. 3
Extreme difficulty.. 4
Stopped doing this because of your eyesight.... 5
Stopped doing this for other reasons or not interested in doing this 6

A5. Because of your eyesight, how much difficulty do you have doing things like shaving, styling your hair, or putting on makeup?

(Circle One)

No difficulty at all.. 1
A little difficulty... 2
Moderate difficulty.. 3
Extreme difficulty.. 4
Stopped doing this because of your eyesight.... 5
Stopped doing this for other reasons or not interested in doing this 6

SUBSCALE: DISTANCE VISION

A6. Because of your eyesight, how much difficulty do you have recognizing people you know from across a room?

(Circle One)

No difficulty at all.. 1
A little difficulty... 2
Moderate difficulty.. 3
Extreme difficulty.. 4
Stopped doing this because of your eyesight.... 5
Stopped doing this for other reasons or not interested in doing this 6

© R 1996

A7. Because of your eyesight, how much difficulty do you have taking part in active sports or other outdoor activities that you enjoy (like golf, bowling, jogging, or walking)?

(Circle One)

No difficulty at all.. 1
A little difficulty... 2
Moderate difficulty... 3
Extreme difficulty.. 4
Stopped doing this because of your eyesight.... 5
Stopped doing this for other reasons or not interested in doing this 6

A8. Because of your eyesight, how much difficulty do you have seeing and enjoying programs on TV?

(Circle One)

No difficulty at all.. 1
A little difficulty... 2
Moderate difficulty... 3
Extreme difficulty.. 4
Stopped doing this because of your eyesight.... 5
Stopped doing this for other reasons or not interested in doing this 6

SUBSCALE: SOCIAL FUNCTION

A9. Because of your eyesight, how much difficulty do you have entertaining friends and family in your home?

(Circle One)

No difficulty at all.. 1
A little difficulty... 2
Moderate difficulty... 3
Extreme difficulty.. 4
Stopped doing this because of your eyesight.... 5
Stopped doing this for other reasons or not

© R 1996

interested in doing this 6

SUBSCALE: DRIVING

A10. [This item, “driving in difficult conditions”, has been included as part of the base set of 25 items as item 16a.]

SUBSCALE: ROLE LIMITATIONS

A11. The next questions are about things you may do because of your vision. For each item, please circle the number to indicate whether for you this is true for you <u>all</u>, <u>most</u>, <u>some</u>, <u>a little</u>, or <u>none</u> of the time.

(Circle One On Each Line)

	All of the time	Most of the time	Some of the time	A little of the time	None of the time
**a. <u>Do you have more help</u> from others because of your vision? ....**	**1**	**2**	**3**	**4**	**5**
**b. <u>Are you limited</u> in the kinds of things you can do because of your vision?**	**1**	**2**	**3**	**4**	**5**

© R 1996

SUBSCALES: WELL-BEING/DISTRESS (#A12) and DEPENDENCY (#A13)

The next questions are about how you deal with your vision. For each statement, please circle the number to indicate whether for you it is definitely true, mostly true, mostly false, or definitely false for you or you don't know.

(Circle One On Each Line)

	Definitely True	Mostly True	Not Sure	Mostly False	Definitely False
**A12. I am often irritable because of my eyesight.**	1	2	3	4	5
**A13. I don't go out of my home alone, because of my eyesight**	1	2	3	4	5

© R 1996
