# Supplementary material for: Patient-Reported Symptoms Versus Clinician-Measured Signs to Distinguish Sjogren's in Patients With Dry Eye
Source: Transl Vis Sci Technol. 2026 Jan 22;15(1):27. doi: 10.1167/tvst.15.1.27 (PMC12849820; doi:10.1167/tvst.15.1.27)
Supplement: Supplement 1 [file tvst-15-1-27_s001.zip › Appendix A VTQ.pdf]

Visual Tasking Questionnaire

Visual Tasking Questionnaire: Suggested Refinement by Modus Outcomes

The following questions ask about how much your **eye condition affected or interfered with your ability to do visual activities in the past 7 days.**

Difficulties doing visual activities might include having to change how you did an activity, needing to take a rest or stop the activity because of your eye condition.

For each question, please choose the answer which describes how difficult it was to do the activity **because of your eye condition** in the past 7 days**.** Please think about **how much difficulty you had in the past 7 days with the visual activities below**.

Over the past 7 days **how difficult was** it to…		**Not at all difficult**	**A little difficult**	**Moderately difficult**	**Very difficult**	**Not applicable**
**1**	Read or type a text message on your cell-phone?	1	2	3	4	
**2**	Look in the bathroom mirror for example to shave or put your make up on?	1	2	3	4	
**3**	Read on paper for example books, newspapers or magazines?	1	2	3	4	
**4**	Read labels or household bills?	1	2	3	4	
**5**	Read on screen for example computer or tablet?	1	2	3	4	
**6**	Watch a program on a screen for example TV or a tablet?	1	2	3	4	
**7**	Work on a computer screen?	1	2	3	4	
**8**	Carry out household chores for example cleaning or laundry?	1	2	3	4	
**9**	Carry out your usual recreational activities or hobbies indoors, such as crafts, sewing, playing cards?	1	2	3	4	
**10**	Watch events at a distance, for example a show or sporting event?	1	2	3	4	

Over the past 7 days **how difficult was** it to…		**Not at all difficult**	**A little difficult**	**Moderately difficult**	**Very difficult**	**Not applicable**
**11**	Locate items in a store when shopping?	1	2	3	4	
**12**	Drive during the day?	1	2	3	4	
**13**	Drive at night?	1	2	3	4	

**16**	What helps you cope most when you have difficulties doing visual activities? (please select all that apply)	☐ Making yourself blink frequently<br>☐ Rubbing or pressing your eyes<br>☐ Resting, or feel the need to rest or close your eyes<br>☐ Stopping the activity because my eyes were too uncomfortable<br>☐ Wearing sunglasses<br>☐ Using eyedrops<br>☐ None of the above
