# Supplementary material for: Patient-Reported Symptoms Versus Clinician-Measured Signs to Distinguish Sjogren's in Patients With Dry Eye
Source: Transl Vis Sci Technol. 2026 Jan 22;15(1):27. doi: 10.1167/tvst.15.1.27 (PMC12849820; doi:10.1167/tvst.15.1.27)
Supplement: Supplement 1 [file tvst-15-1-27_s001.zip › Appendix A CESD.pdf]

Center for Epidemiologic Studies Depression Scale (CES-D), NIMH

Below is a list of the ways you might have felt or behaved. Please tell me how often you have felt this way during the past week.

	During the Past Week			
	Rarely or none of the time (less than 1 day)	Some or a little of the time (1-2 days)	Occasionally or a moderate amount of time (3-4 days)	Most or all of the time (5-7 days)
1. I was bothered by things that usually don't bother me.	☐	☐	☐	☐
2. I did not feel like eating; my appetite was poor.	☐	☐	☐	☐
3. I felt that I could not shake off the blues even with help from my family or friends.	☐	☐	☐	☐
4. I felt I was just as good as other people.	☐	☐	☐	☐
5. I had trouble keeping my mind on what I was doing.	☐	☐	☐	☐
6. I felt depressed.	☐	☐	☐	☐
7. I felt that everything I did was an effort.	☐	☐	☐	☐
8. I felt hopeful about the future.	☐	☐	☐	☐
9. I thought my life had been a failure.	☐	☐	☐	☐
10. I felt fearful.	☐	☐	☐	☐
11. My sleep was restless.	☐	☐	☐	☐
12. I was happy.	☐	☐	☐	☐
13. I talked less than usual.	☐	☐	☐	☐
14. I felt lonely.	☐	☐	☐	☐
15. People were unfriendly.	☐	☐	☐	☐
16. I enjoyed life.	☐	☐	☐	☐
17. I had crying spells.	☐	☐	☐	☐
18. I felt sad.	☐	☐	☐	☐
19. I felt that people dislike me.	☐	☐	☐	☐
20. I could not get "going."	☐	☐	☐	☐

SCORING: zero for answers in the first column, 1 for answers in the second column, 2 for answers in the third column, 3 for answers in the fourth column. The scoring of positive items is reversed. Possible range of scores is zero to 60, with the higher scores indicating the presence of more symptomatology.
