# Supplementary material for: Patient-Reported Symptoms Versus Clinician-Measured Signs to Distinguish Sjogren's in Patients With Dry Eye
Source: Transl Vis Sci Technol. 2026 Jan 22;15(1):27. doi: 10.1167/tvst.15.1.27 (PMC12849820; doi:10.1167/tvst.15.1.27)
Supplement: Supplement 1 [file tvst-15-1-27_s001.zip › Appendix A ESSPRI.pdf]

EULAR Sjogren's Syndrome Patient Reported Index (ESSPRI)

1. How severe has your dryness been during the last 2 weeks?

	0	1	2	3	4	5	6	7	8	9	10	
No dryness	☐	☐	☐	☐	☐	☐	☐	☐	☐	☐	☐	Maximal imaginable dryness

2. How severe has your fatigue been during the last 2 weeks?

	0	1	2	3	4	5	6	7	8	9	10	
No fatigue	☐	☐	☐	☐	☐	☐	☐	☐	☐	☐	☐	Maximal imaginable fatigue

3. How severe has your pain (joint or muscular pains in your arms or legs) been during the last 2 weeks?

	0	1	2	3	4	5	6	7	8	9	10	
No pain	☐	☐	☐	☐	☐	☐	☐	☐	☐	☐	☐	Maximal imaginable pain
