# Supplementary material for: Patient-Reported Symptoms Versus Clinician-Measured Signs to Distinguish Sjogren's in Patients With Dry Eye
Source: Transl Vis Sci Technol. 2026 Jan 22;15(1):27. doi: 10.1167/tvst.15.1.27 (PMC12849820; doi:10.1167/tvst.15.1.27)
Supplement: Supplement 1 [file tvst-15-1-27_s001.zip › Appendix A IDEEL.pdf]

Impact of Dry Eye on Everyday Life (IDEEL) Questionnnaire

Daily Activities

- The following is a list of day-to-day activities that you may or may not have participated in **<u>OVER</u>**
<u>THE LAST TWO WEEKS</u>.
- If you participated in the activity, please choose how often you were **limited in or stopped doing** the activity **<u>BECAUSE OF YOUR DRY EYES</u>**.
- Choose 'I did not perform this activity due to reasons OTHER than dry eye' if you did not take part in the activity for reasons other than your dry eyes.
- Please choose only one box per question.

**<u>OVER THE LAST TWO WEEKS</u>**, how limited were you in doing the following activities **<u>BECAUSE OF YOUR DRY EYES</u>**?	I did not do this activity for reasons **OTHER** than my dry eyes/Not applicable	**<u>OVER THE LAST TWO WEEKS</u> I was limited <u>BECAUSE OF MY DRY EYES</u>:** None of the time (5)	A little of the time (4)	Some of the time (3)	Most of the time (2)	All of the time (1)	I can **no longer** do this activity **due to my dry eyes** (0)
1. Doing close work in the **morning or afternoon** (such as crossword puzzles, reading, looking at a computer, and/or sewing)	☐	☐	☐	☐	☐	☐	☐
2. Doing close work in the **evening or at night**	☐	☐	☐	☐	☐	☐	☐
3. Driving	☐	☐	☐	☐	☐	☐	☐
4. Being around and/or using scented products (such as cologne or hairspray)	☐	☐	☐	☐	☐	☐	☐
5. Working on a computer	☐	☐	☐	☐	☐	☐	☐
6. Going somewhere where there is tobacco smoke or being around someone who smokes	☐	☐	☐	☐	☐	☐	☐

7. Wearing contact lenses	☐	☐	☐	☐	☐	☐	☐
8. Wearing make-up near or on my eyes	☐	☐	☐	☐	☐	☐	☐
9. Flying on an airplane	☐	☐	☐	☐	☐	☐	☐

Feelings

The following questions ask about how dry eye affected your mood and social life **OVER THE LAST TWO WEEKS**. Please choose how **often** you experienced each **feeling BECAUSE OF YOUR DRY EYES**. Please choose only one box per question.

**OVER THE LAST TWO WEEKS**, how often do you have each of the following feelings **BECAUSE OF YOUR DRY EYES**?	**OVER THE LAST TWO WEEKS**, I experienced this feeling **BECAUSE OF MY DRY EYES**:				
	None of the time (4)	A little of the time (3)	Some of the time (2)	Most of the time (1)	All of the time (0)
10. Irritability	☐	☐	☐	☐	☐
11. Impatience	☐	☐	☐	☐	☐
12. Feeling sad	☐	☐	☐	☐	☐
13. Worry that my dry eyes will get worse	☐	☐	☐	☐	☐
14. Feeling annoyed	☐	☐	☐	☐	☐
15. Feeling like my eyes do not look nice	☐	☐	☐	☐	☐
16. Feeling like I have to make adjustments to my life	☐	☐	☐	☐	☐
17. Feeling different from other people because of my dry eyes	☐	☐	☐	☐	☐
18. Feeling like I am always aware of my eyes	☐	☐	☐	☐	☐
19. Feeling older than I really am	☐	☐	☐	☐	☐

20. Feeling like people look at me and think I am fine when I'm not	☐	☐	☐	☐	☐
21. Feeling like there is nothing I can do for my dry eyes	☐	☐	☐	☐	☐

Work

22. Are you currently working?

- ☐ Yes If "Yes," please read the instructions below.
- ☐ No

The following questions ask about how often your dry eyes affected your work life **OVER THE LAST TWO WEEKS**. Please choose how **often** you experienced the following situations **at work BECAUSE OF YOUR DRY EYES**. Please choose only one box per question.

	**OVER THE LAST TWO WEEKS** I experienced the following situation at work **BECAUSE OF MY DRY EYES:**				
**OVER THE LAST TWO WEEKS**, how often did you experience the following situations at work **BECAUSE OF YOUR DRY EYES**?	None of the time (4)	A little of the time (3)	Some of the time (2)	Most of the time (1)	All of the time (0)
23. Feeling distracted	☐	☐	☐	☐	☐
24. Feeling like I couldn't concentrate	☐	☐	☐	☐	☐
25. Having to take a break from work	☐	☐	☐	☐	☐
26. Having to change the way I work (such as the way I read, look at a computer, or work outside)	☐	☐	☐	☐	☐
27. Having to change my work environment (such as how close I am to an air conditioning or heating vent)	☐	☐	☐	☐	☐

Thank you for finishing this questionnaire.
Please make sure that you answered every question.

Symptom Bother

These questions ask about the symptoms you may experience due to dry eyes.

1. **__OVER THE LAST TWO WEEKS__**, how often did you experience dry eye symptoms?

None of the time	A little of the time	Some of the time	Most of the time	All of the time
☐	☐	☐	☐	☐

The following questions ask about how bothersome dry eye symptoms were to you **__OVER THE LAST TWO WEEKS__**. If you had the symptom, please choose **how much the symptom bothered you** (not at all, slightly, moderately, or very much). If you did not have the symptom over the last two weeks, choose the "I did not have this symptom / Not applicable" box. Please choose only one box per question.

**__OVER THE LAST TWO WEEKS__**, how much did each of the following symptoms bother you?	I did not have this symptom / Not applicable	**__OVER THE LAST TWO WEEKS__, I had this symptom and it bothered me:** Not at all	Slightly	Moderately	Very much
2. Eyes that felt gritty or sandy	☐	☐	☐	☐	☐
3. Felt like I needed to close my eyes even though I was not tired	☐	☐	☐	☐	☐
4. Burning or stinging eyes	☐	☐	☐	☐	☐
5. Tired eyes	☐	☐	☐	☐	☐
6. Blurry vision	☐	☐	☐	☐	☐
7. Itchy eyes	☐	☐	☐	☐	☐
8. Irritated eyes	☐	☐	☐	☐	☐
9. Eyes that felt like they had been scratched by something	☐	☐	☐	☐	☐

10. Eye dryness	☐	☐	☐	☐	☐

**<u>OVER THE LAST TWO WEEKS</u>**, how much did each of the following symptoms bother you?	I did not have this symptom / Not applicable	**<u>OVER THE LAST TWO WEEKS</u>, I had this symptom and it bothered me:**			
		Not at all	Slightly	Moderately	Very much
11. Mucus in, around, or coming out of my eyes	☐	☐	☐	☐	☐
12. Puffy or swollen eyes	☐	☐	☐	☐	☐
13. Eye redness	☐	☐	☐	☐	☐
14. Aching or sore eyes	☐	☐	☐	☐	☐
15. Felt like something was in my eye	☐	☐	☐	☐	☐
16. Frequent and/or rapid blinking	☐	☐	☐	☐	☐
17. Difficulty blinking because of little or no moisture in my eyes	☐	☐	☐	☐	☐
18. Sensitivity to light, glare, and/or wind	☐	☐	☐	☐	☐
19. Sensitivity to recirculated air (such as air conditioning and heat)	☐	☐	☐	☐	☐
20. Headaches associated with dry eye symptoms	☐	☐	☐	☐	☐

Thank you for finishing this questionnaire.
Please make sure that you answered every question.

Treatment - In General

People use many different types of treatment for dry eyes, including eye drops, ointments, punctal plugs and/or eye masks.

1. **<u>OVER THE LAST TWO WEEKS,</u>** how often did you use treatment for your dry eyes?

None of the time	A little of the time	Some of the time	Most of the time	All of the time
☐ (please skip to question 7 on the next page)	☐ (please read the instructions below)	☐ (please read the instructions below)	☐ (please read the instructions below)	☐ (please read the instructions below)

The following questions ask about **all** the **dry eye** treatments you used **<u>OVER THE LAST TWO WEEKS</u>**. Please choose how **often** the following statements are **true**. Please choose only one box per question.

**<u>OVER THE LAST TWO WEEKS</u>**, how often were the following statements **<u>TRUE</u>**?	None of the time	A little of the time	Some of the time	Most of the time	All of the time
2. I was happy with how quickly my treatments worked	☐	☐	☐	☐	☐
3. I was happy with how long the effects of my treatments lasted	☐	☐	☐	☐	☐
4. The treatments I used **<u>completely eliminated</u>** my dry eye symptoms	☐	☐	☐	☐	☐
5. The treatments I used **<u>relieved most</u>** of my dry eye symptoms	☐	☐	☐	☐	☐
6. I was bothered by how often I had to use dry eye treatments	☐	☐	☐	☐	☐

Treatment – Eye Drops

7. Do you ever use eye drops to treat your dry eyes?

- ☐ Yes If "Yes," please read the instructions below.
- ☐ No

The following questions ask specifically about **eye drops**. Please choose an answer that represents how **often** you experienced the following situations **OVER THE LAST TWO WEEKS**. Please choose only one box per question.

**OVER THE LAST TWO WEEKS**, how often did you experience the following situations?	None of the time	A little of the time	Some of the time	Most of the time	All of the time
8. I was bothered by blurriness shortly after using my eye drops	☐	☐	☐	☐	☐
9. I was embarrassed when I had to use eyedrops	☐	☐	☐	☐	☐
10. I felt like I could not go anywhere without my eye drops	☐	☐	☐	☐	☐

Thank you for finishing this questionnaire.
Please make sure that you answered every question.
