# Supplementary material for: Patient-Reported Symptoms Versus Clinician-Measured Signs to Distinguish Sjogren's in Patients With Dry Eye
Source: Transl Vis Sci Technol. 2026 Jan 22;15(1):27. doi: 10.1167/tvst.15.1.27 (PMC12849820; doi:10.1167/tvst.15.1.27)
Supplement: Supplement 1 [file tvst-15-1-27_s001.zip › Appendix A Mood.pdf]

Mood Questionnaire

We will now ask you several questions about your mood and how you've been feeling. Circle the number in the box that best represents each answer.

		Not at all	No more than usual	Rather than usual	Much more than usual
**MQ 1**	**Have you been thinking of yourself as a worthless person?**	0	1	2	3
**MQ 2**	**Have you felt that life is entirely hopeless?**	0	1	2	3
**MQ 3**	**Have you felt that life isn't worth living?**	0	1	2	3
**MQ 4**	**Have you thought of the possibility that you might make away with yourself?**	0	1	2	3
**MQ 5**	**Have you found at times you couldn't do anything because your nerves were too bad?**	0	1	2	3
**MQ 6**	**Have you found yourself wishing you were dead and away from it all?**	0	1	2	3
**MQ 7**	**Have you found that the idea of taking your own life kept coming into your mind?**	0	1	2	3
